# Antibacterial ability and hemocompatibility of graphene functionalized germanium

**DOI:** 10.1038/srep37474

**Published:** 2016-11-23

**Authors:** Hao Geng, Jiayun Dai, Jinhua Li, Zengfeng Di, Xuanyong Liu

**Affiliations:** 1 State Key Laboratory of High Performance Ceramics and Superfine Microstructure, Shanghai Institute of Ceramics, Chinese Academy of Sciences, Shanghai 200050, China; 2 State Key Laboratory of Functional Materials for Informatics, Shanghai Institute of Microsystem and Information Technology, Chinese Academy of Sciences, Shanghai 200050, China; 3 University of Chinese Academy of Sciences, Beijing 100049, China

## Abstract

Germanium (Ge), as an elemental semiconductor material, has been an attractive candidate for manufacturing semiconductor microelectronic device. In the present investigation, to improve the biocompatibility of Ge-based device, graphene film is directly deposited on the Ge surface with different coverage area by controlling the growth time. Compared to bare Ge, the presence of graphene film entitles Ge with satisfactory antibacterial ability against *Staphylococcus aureus (S.aureus*), and acceptable antibacterial ability against *Escherichia coli (E. coli*). Meanwhile, antibacterial efficiency closely correlates with coverage area of graphene film, and larger graphene coverage always leads to better antibacterial performance. The underlying mechanism is thought to be the integrative action of phospholipids disturbance and electron extraction at the interface between graphene and biomembrane. Meanwhile, the electron extraction action would further lead to the activation of platelet. This study might provide some new insights into the relationship between antibacterial ability and hemocompatibility based on graphene functionalized biomedical device.

The rapid developments of biotechnology, biomaterials and tissue engineering have resulted in advances in medical diagnostics. Of particular significance are the bio-electronic devices (e.g., biochips, biosensors and MEMS). Nevertheless, the biocompatibility is always a crucial issue which limits the feasibility and longevity of the implantable bio-electronic device^1^. Therefore, some attempts have been made to improve the bioactivity and biocompatibility of the materials for the implantable device^2^,^3^,^4^. As an indispensible material of microelectronic device, Ge has undergone a resurgence as research communities’ appeals for higher carrier mobility to replace the mainstream silicon (Si) in the past decades^5^,^6^. Moreover, single-crystal Ge has been considered as an ideal material for constructing thermal imaging systems attribute to its transparency in infrared band^7^. These properties have made Ge an attractive candidate for microelectronic device^8^. However, the instability of surface germanium oxide layer, which is necessary for current planar device technology, has impeded the wide application of Ge-based device^9^.

Considering the sensibility of physiological environment, biomaterials and biochips remain subjected to the host immune response against foreign bodies^2^. Without favorable biocompatibility, implanted devices would lead to tissue injury and inflammatory reaction^10^. Meanwhile, surface character is responsible for the biocompatibility of implanted device. Therefore, one of the effective ways to relieve/eliminate the biocompatibility problem and further to promote beneficial biological effects is to design bioactive layer on the surface of implanted devices^3^,^4^,^11^. For the sake of exploring potential application of Ge-based bio-electronic device, suitable surface treatment appears to be of particular importance for meeting the clinical needs.

Graphene, as a single-atom-thick sheet of sp^2^ hybridized carbon atoms packed in two-dimensional honeycomb structure, has been one of the most fascinating nano-materials ascribed to its exceptional physical and chemical performance^12^. In recent years, it has been reported that chemically exfoliated graphene oxide, reduced graphene oxide as well as chemical vapor deposited (CVD) graphene present favorable cytocompatibility, which enables the application potential of graphene as medical implanted materials^13^,^14^,^15^,^16^. With the advantage of large area, smooth and few defects, graphene films grown by CVD approach show excellent abilities to promote cell adhesion and stem cell differentiation^15^,^16^,^17^. Nevertheless, the intrinsic characteristics of graphene can be significantly affected by the size, substrate types and even deposited temperature^17^,^18^,^19^,^20^. Researching on these points will help identify the internal relationship and further illuminate the underlying mechanism.

Our previous works have proved that relatively perfect monolayer graphene films can be directly deposited on Ge surface by CVD^20^,^21^,^22^. In this work, graphene films with different coverage area are deposited on the Ge surface by controlling the growth time. Through systematic tests including the corrosion resistance, anti-inflammatory ability and hemocompatibility, this paper is try to explore the application potential of Ge as biochips and biosensors by overriding it with graphene films.

## Results

### Characterization of graphene coated Ge substrate

**Figure 1:**
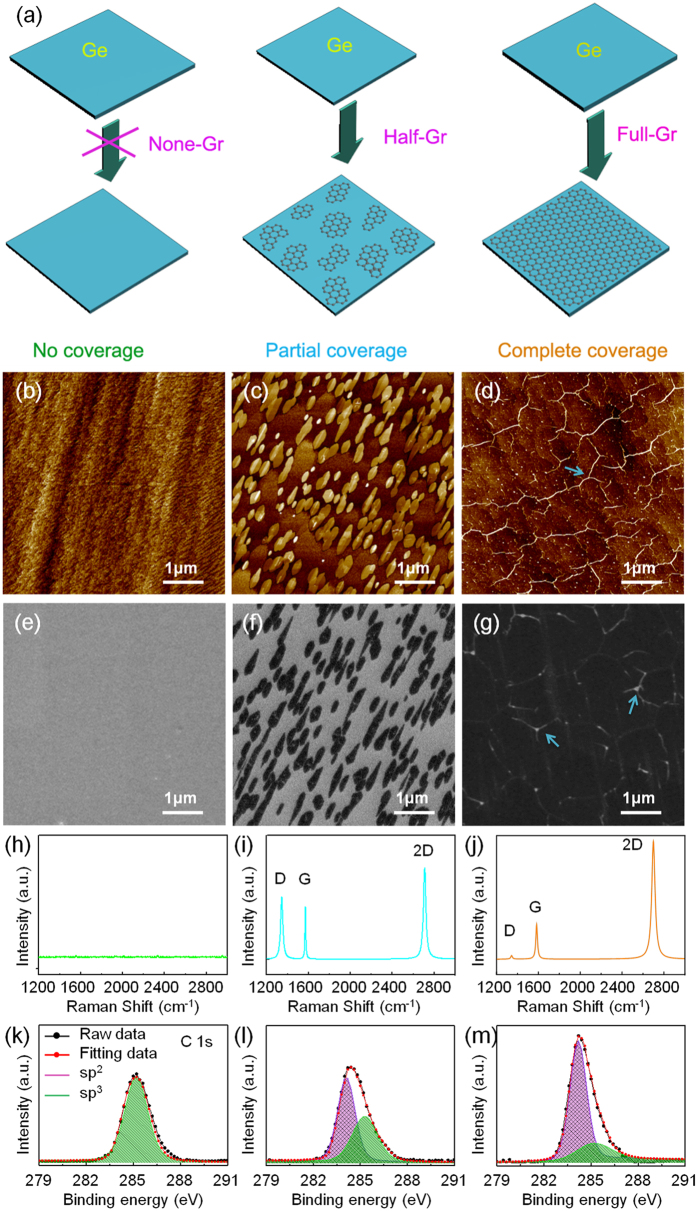
First panel (a): Schematic fabrication processes corresponding to three typical graphene covered Ge substrates with different coverages, i.e., bare Ge substrate (None-Gr), partially covered Ge substrate (Half-Gr) and fully covered substrate (Full-Gr); second panel: (b–d) AFM morphologies of None-Gr (**b**), Half-Gr (**c**) and Full-Gr (**d**) samples, respectively; third panel: (e–g) SEM morphologies of None-Gr (**e**), Half-Gr (**f**) and Full-Gr (**g**) samples, respectively; fourth panel: (h–j) Raman spectra of None-Gr (**h**), Half-Gr (**i**) and Full-Gr (**j**) samples; bottom panel: (k–m) XPS measurements of None-Gr (**k**), Half-Gr (**l**) and Full-Gr (**m**) samples; the blue arrows in (**d**) and (**g**) indicate the wrinkle.

The graphene films are deposited on the Ge(110) substrate by atmospheric pressure chemical vapor deposition (APCVD). Figure 1a shows the schematic fabrication processes corresponding to three typical graphene coated Ge substrates with different coverage area, i.e., bare Ge substrate, partially covered Ge substrate and fully covered substrate. And, their corresponding AFM and SEM images are shown in Fig. 1a–g. Figure 1b displays a typical Ge surface without graphene coverage, and an atomically flat surface separated by 0.2–0.4 nm high atomic steps. After grown for 100 min, a large number of graphene domains with a few hundred nanometer size can be observed and the Ge surface is about 50% covered by graphene, as shown in Fig. 1c and f. When increasing the growth time to 200 min, the graphene domains expand and merge to form a continuous film with wrinkled structures, as displayed in Fig. 1d and g (indicated by blue arrows). In the following section, they are marked as None-Gr, Half-Gr and Full-Gr for the bare Ge substrate, partial-covered and complete-covered Ge substrate, respectively. The Raman spectrum of graphene consisting of three major features, the G peak (~1580 cm^−1^), the 2D peak (~2700 cm^−1^) and the defect related D peak (1350 cm^−1^), is shown in Fig. 1h–j 23,24. A large D peak shown in Fig. 1i results from the domain edges, which exist as the individual small graphene domain and unable to merge with the other one. When the growth time extends to 200 min, no appreciable D peak and a high intensity (I_2D_/I_G_) above 3 is observed in Fig. 1j, indicating all domain edges disappear due to the seamless mergence of the graphene domains. In addition, the Raman mapping in Figure S1 also suggests the formation of the continuous graphene film on Ge(110) surface. XPS measurement is also used to investigate the chemical structure evolution of the various elements on Ge(110) surface. For bare Ge substrate (Fig. 1k), the C 1 s spectra consists a carbon sp^3^-hybridization peak located at 285.1 eV, which comes from natural carbon contamination^25^. After the growth of graphene for 100 min, a presence of sp^2^-hybridization peak at 284.6 eV as well as a slight decrease in the intensity of the sp^3^ peak can be observed in Fig. 1l, indicating the formation of small graphene domains on the Ge(110) surface. With increasing the growth time to 200 min, sp^2^ hybridization of the C 1 s spectra dominates (Fig. 1m), suggesting the formation of a continuous graphene film, which is consistent with AFM and SEM measurements.

### Corrosion resistance of graphene coated Ge substrate

**Figure 2:**
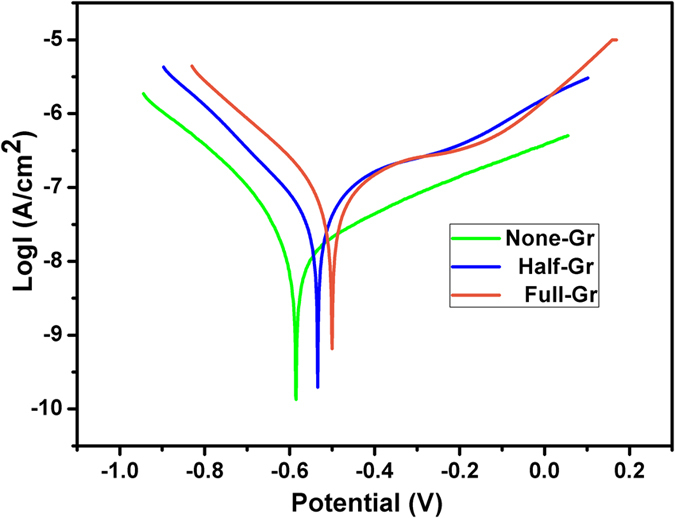
Potentiodynamic polarization curves of None-Gr, Half-Gr and Full-Gr in 0.9% physiological saline solution.

Corrosion resistance is one of the significant factors to be considered in course of practical application of biomedical devices. In order to evaluate the impact of the graphene coverage area on the corrosion resistance of the underlying Ge, the dynamic potential curve test was conducted in the physiological saline (0.9 wt% NaCl solution). As depicted in Fig. 2, the corrosion potential shows distinct positive shift for both Half-Gr and Full-Gr compared to the None-Gr, which indicates a lower corrosion tendency for graphene covered ones. Moreover, it can be found that Ge substrate covered by the continuous graphene film exhibits better corrosion resistance compared to that covered by the partial graphene film. Therefore, it is concluded that graphene film can act as a protective layer for Ge-based device in corrosion medium.

### Bacteria response to graphene coated Ge substrate

**Figure 3:**
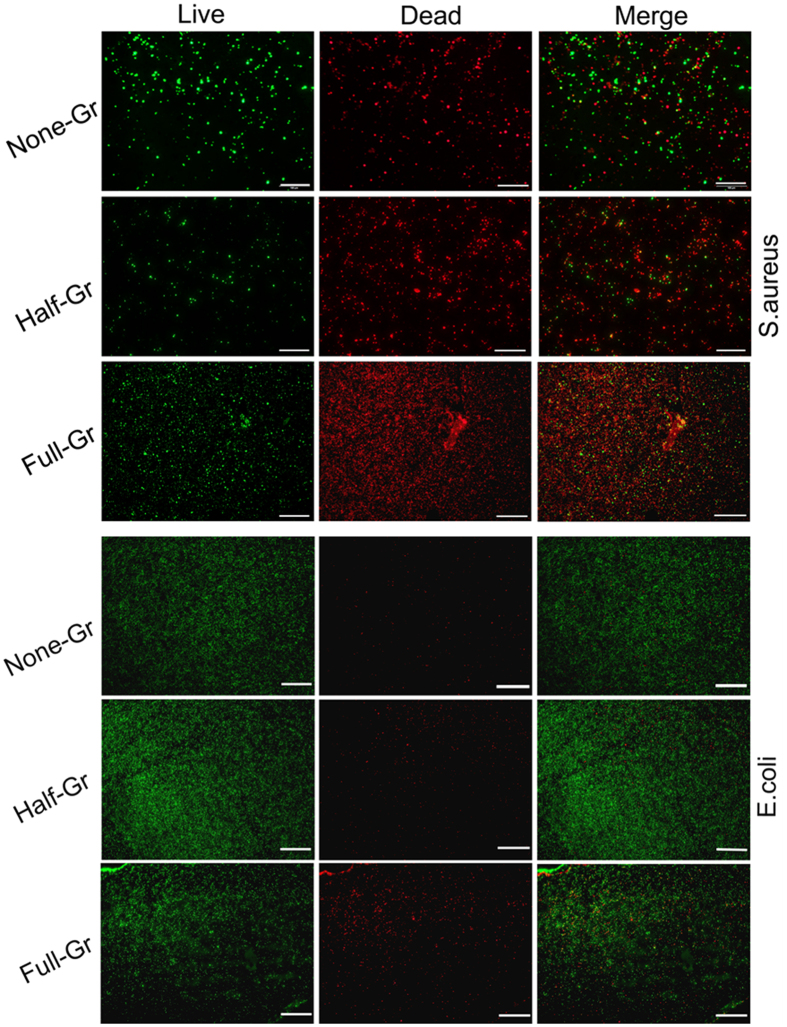
(**a** and **b**) Representative fluorescence images showing the bacteria viability and membrane integrity of *S.aureus* (**a**)/*E. coli* (**b**) on the surface with variable coverage area of graphene films after incubating for 24 h, visualized by staining with LIVE/DEAD^®^ BacLight™ Bacterial Viability Kit. Living bacteria with intact membrane show green fluorescence while dead ones with disturbed membrane present red fluorescence. Scale bar: 100 μm.

Gram-positive *Staphylococcus aureus (S.aureus*) and Gram-negative *Escherichia coli (E. coli*) are used as model bacterium to inoculate on the surface of graphene coated Ge substrate. In order to better visualize the interactive behaviors of bacteria when contacting with graphene covered Ge substrate, Live/Dead Kit is utilized for bacteria fluorescent staining. As shown in Fig. 3, the increment of graphene film coverage area is able to boost the number of adhered living *S.aureus* significantly. Meanwhile, the damaged bacteria display even more remarkable tendency, and observation vision is filled with intense red fluorescence derived from the listless and dead bacteria due to the damaged cell membrane, as depicted in the middle panel. However, the obvious increase in the proportion of the dead bacteria is observed on the Half-Gr where the surface is covered with graphene films, although not completely covered. Furthermore, massive damaged bacteria fill up the whole surface of Full-Gr. However, in case of *E. coli*, the red spots which refer to the dead bacteria (in web version) on the None-Gr are barely observed, while the surface is occupied by green spots, which refer to the living bacteria (in web version), indicating the inability of None-Gr to inactivate *E. coli*. The dead bacteria are hard to be observed on Half-Gr either (a very small amounts of dead bacteria can be observed), and only appear on Full-Gr with trace amount. Therefore, compared to *E. coli*, *S.aureus* is more susceptible to the presence of graphene coverage on Ge substrate.

In addition, the bacterium solution is re-inoculated on the agar plate after detaching from the surface of incubated specimen to further incubation for 16 h, and the typical photographs is depicted in Figure S2. Obviously, re-cultivated colonies of *S.aureus* and *E. coli* which are detached from the surface of the None-Gr are observed on the agar plate. However, when the bacteria contact with the Half-Gr surface, the number of *S.aureus* colonies reduces significantly, while the number of *E. coli* colonies remains high. In addition, the number of *S.aureus* colonies continues to decrease on the Full-Gr. Meanwhile, the number of *E. coli* colonies which are detached from Full-Gr ones also present a partially decrease compared to that from the Half-Gr ones. The result of reduction of Alamar blue in Figure S3 shows a well consistence with the result of agar culture plate.

**Figure 4:**
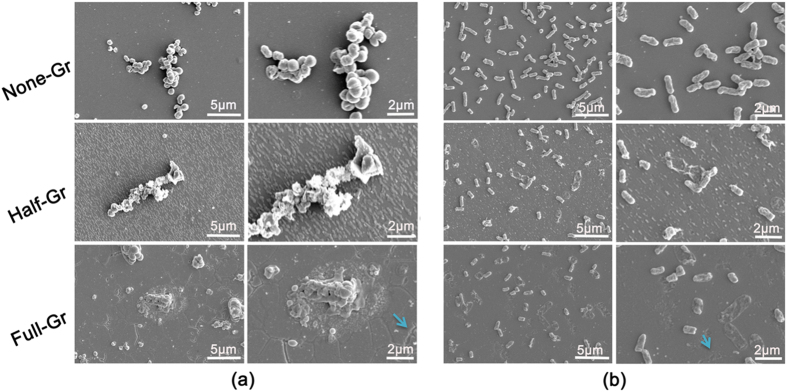
(**a** and **b**) Representational SEM morphology of *S.aureus* (**a**) and *E. coli* (**b**) adhered on the surface with variable coverage area of graphene films after incubating for 24 h at both low (left panel) and high magnification (right panel), blue arrows indicate the crack and fracture.

To further confirm the influence of the graphene coverage on the bacteria phenotype, SEM is utilized to inspect the membrane integrity and morphology of the two distinct bacteria, as displayed in Fig. 4. *S.aureus* on the None-Gr surface basically maintains original spherical appearance whereas those on the Half-Gr surface are disintegrated with severely distorted shape. Furthermore, the glomerate bacteria on the Full-Gr surface exhibit apparently deathlike state due to the crack of membrane. Meanwhile, the evident residue of exudative cytoplasm appears around the bacteria swarm. The similar manner is also observed for *E. coli*. On the None-Gr surface, the bacteria keep the typical rod-like shape. When the bacteria contact with Half-Gr surface, a minority of bacteria behaves with disrupted shape and loses the integrity of cell membrane while the majority of bacteria still remain the intrinsic shape. Nevertheless, the bacteria on the Full-Gr show irregular appearance characteristics, some bacteria have totally lost the cytoplasm ascribe to the fracture of membrane, which indicates poor living state. And, some robust bacteria with intact membrane still exist as well.

### Hemocompatibility of graphene coated Ge substrate

**Figure 5:**
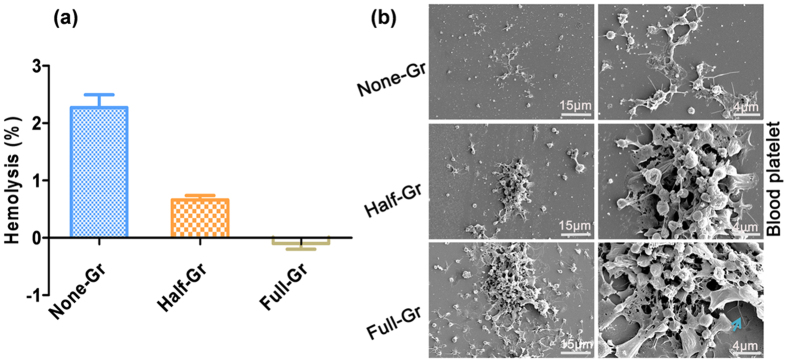
(**a**) Hemolysis rate vs the graphene coverage when contacting with fresh blood; (**b**) Typical blood platelet adhesion morphology on the surface with variable graphene coverages area after incubating for 1 h at low (left panel) and high magnification (right panel), blue arrow indicate the wrinkle.

Infaust interface interaction between red blood cells and biomaterials in the physiological saline has the opportunity to give rise to the leakage of intercellular hemoglobin, which indicates the occurrence of hemolysis. According to the ISO 10993-4, biomaterials with the hemolysis rate less than 5% can be considered as feasible blood-contacting materials. As shown in Fig. 5a, None-Gr presents a higher hemolysis rate compared to Half-Gr and Full-Gr ones. With increase of the graphene coverage, the hemolysis rate tends to decrease. Figure 5b presents the platelet adhesion morphology on the None-Gr, Half-Gr and Full-Gr surface. It can be observed that intrinsic spherical shape of platelet has been activated and become dendritical shape on the None-Gr surface with the pseudopodium stretched from the edge of the platelet. Moreover, the aggregation of activated platelets is not obvious. As for the Half-Gr specimen, the adherent platelets with increased amount create the proper conditions for platelet activation and aggregation. More strikingly, both the amount and the activation rate of platelets are significantly enhanced on the surface of Full-Gr compared to that of Half-Gr ones. In addition, the aggregation of activated platelet became more remarkable.

## Discussion

For the past few years, the antibacterial mechanism of graphene and its derivates proposed by previous works mainly focus on physical damage caused by piercing through the bacteria membrane due to the sharp edge structure of graphene^26^. Obviously, this kind of physical interaction mechanism is no longer applicable to the CVD-grown graphene and microbe system because the graphene film synthesized by CVD approach is homogeneously smooth, which is distinct from the GO and rGO sheets. The destruction of bacteria membrane stems from graphene induced oxidative stress has been reported by other research communities^27^,^28^. Moreover, experimental and theoretical study show that strong dispersion interaction between graphene and phospholipids eventually lead to the membrane degradation by robust extraction of phospholipids^29^,^30^. Meanwhile, with the advantage of presence of dirac point, graphene can be assumed as a semimetal, and it would lead to the forming of Schottky barrier at the interface when contact with a semiconductor^20^,^21^. In this work, graphene layer has been deposited on the slightly p-type doping Ge. As a result, the surface of graphene tends to be positively charged and hydrophobic (Figure S4) arise from reverse flow of holes and electrons. Electrostatic interaction between positively charged pristine graphene and negatively charged bacterial membrane makes the phospholipids band together with graphene films. Meanwhile, the contact might induce translocation and overturn of phospholipids and make phospholipids interact with hydrophobic tail due to hydrophobic interaction, which further disrupt the function of the membrane^30^. Therefore, the bacteria death caused by membrane rupture would be very likely to occur, since the translocation of phospholipids is inclined to destroy the structure of bacterial membrane. For this reason, it can be speculated that Half-Gr ones may have an inferior hydrophobic interaction performance compared to the Full-Gr ones, which is supported by the contact angle test (Figure S4). With respect to the difference of antibacterial efficiency for *S.aureus* and *E. coli* at the same conditions, this could be due to the bacteria structure difference where the outer film of *E. coli* protects itself against extracellular perturbation, whereas *S.aureus* has no outer film^26^. Therefore, phospholipids perturbation induced membrane damage by graphene may be the part reason that gives rise to the bacteria death.

**Figure 6:**
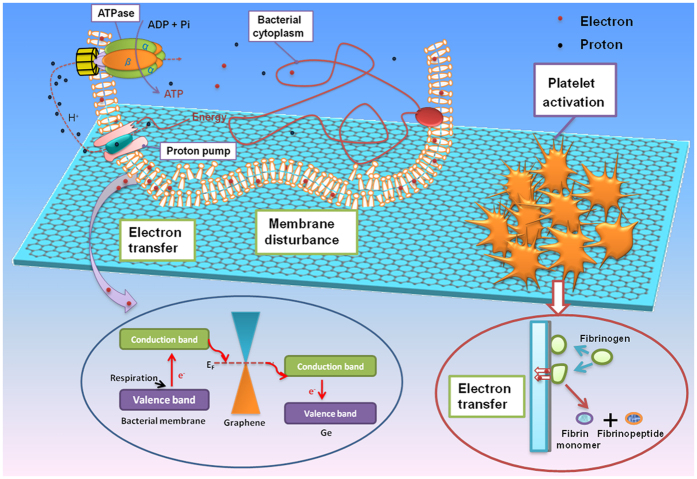
Schematic drawing of interaction between bacteria (blood platelet) and graphene films covered Ge substrate. The interaction mainly focus on two parts: 1) electron transfer process from the bacterial membrane (fibrinogen) to graphene and then to the Ge substrate; 2) membrane disturbance ascribed to the hydrophobic force.

However, considering the outstanding electronic properties of graphene ascribed to the delocalized π bond, electron transfer between graphene and microbe seems to be possible through respiratory action^21^,^31^. Thus, graphene can be regarded as extracellular electron acceptor with the ability to realize charge transport and carrier separation^32^,^33^. According to endosymbiotic theory, a microbial cell is similar to the mitochondrion of a mammalian cell. Furthermore, electron transfer conduit based on respiratory proteins exists between bacteria and extracellular environment^34^. Therefore, the conductivity of substrate plays a crucial role in modulating the bacteria behaviors. Graphene possesses metallicity at a sufficient Fermi energy, and obviously, a Schottky barrier will form at the interface between graphene and Ge semiconductor. Our previous works have investigated the conductivity of graphene/Ge substrate junction along with the change of substrate conductivity as well as the crystallinity-dependent graphene/Ge junction^20^,^21^. The *I-V* characteristic curve (Figure S5) indicates that graphene@Ge behaves as a typical Schottky diode with rectifying behavior. On the other hand, microbe always possesses a negative resting membrane potential ranging from −200 mV to −20 mV and the respiratory protein may possess semiconductivity with a band gap of 2.6 eV to 3.1 eV^35^. Therefore, electrons readily transfer from microbial membrane to graphene, while graphene behaves like a bridge to facilitate electron transferring to the graphene@Ge Schottky barrier which can act as electron trap. So, the conduit is presumed to exist for electron transfer between bacteria and extracellular environment. To illustrate the electron transfer process, some available data are collected and used to draw the schematic diagram, as presented in Fig. 6 36,37. As discussed above, graphene is used as extracellular electron receptor by bacteria to perform respiration function, but the respiratory electrons was captured by graphene irreversibly, thus perturbing the membrane physiological function. With the full coverage of graphene, the Full-Gr possesses superior electron extracting and trapping capability than the defective Half-Gr, thus leading to better antibacterial ability. In addition, the lower isoelectric point of *S.aureus* membrane compared to that of *E. coli* results in a more negatively charged surface, which may further lead to the resistance difference against extracellular environment^38^. Meanwhile, the location for gram-negative bacteria to produce energy by electron transport system lies in the inner cytoplasmic membrane^39^, so it may be more difficult for graphene to extract electron from *E. coli* than *S.aureus*.

Besides the antibacterial performance, our results also show that CVD-graphene covered Ge substrate would induce remarkable platelet adhesion and activation. Meanwhile, the larger coverage area of graphene film apparently results in more adherent and activated platelets. In fact, when biomaterials come in contact with platelets, protein absorption will occur firstly^40^. Hydrophobic surface would absorb more proteins from plasma (especially for fibrinogen) and subsequently induce the conformation changes of the absorbed proteins^41^. Meanwhile, positive surface charges often facilitate the protein adsorption through electrostatic interactions and stimulate platelet adhesion and activation^42^. The noncovalent interactions between graphene and biomacromolecule through π-π bond will promote protein adhension on the graphene surface. Fibrinogen, an important protein associated with coagulation, is deemed to have a structure similar to the intrinsic semiconductor. When contacting with heterogonous material, the electron transfer process from occupied valence band of fibrinogen to free state of the materials presumably happened, which will contribute to the decomposition and distortion of the protein^40^,^43^. So it can be easy to speculate that graphene can act as an electron acceptor and transporter, and build a bridge between fibrinogen and graphene/Ge junction subsequently (as shown in Fig. 6). As a result, the loss of electron will lead to the change of the conformation of fibrinogen. Along with the subsequent formation of fibrinopeptide and fibrin monomer, coagulation process is further activated. More activated platelet would be found on the Full-Gr surface compared to the Half-Gr ones because of the superior electron extraction ability. Thus, by taking advantage of favourable antibacterial property and electron extraction ability, CVD-graphene covered Ge substrates are anticipated to be utilized as the underlying hemostatic materials. In addition, they also possess the potential for constructing thrombin protein detection biosensors.

In summary, graphene films can act as a qualified protective film for Ge substrate to resist corrosion and the presence of graphene is able to significantly alter the antibacterial capability of Ge substrate. Moreover, Ge substrate with the continuous coverage of graphene exhibits remarkable antibacterial ability. The integrative action of phospholipids disturbance and electron extraction at the interface between graphene and biomembrane are believed to be responsible for the improved antibacterial performance. In addition, the electron extraction action would further promote the activation of platelets. The surface and interfacial reactions which concentrated on electron transfer process may provide new insights for the graphene functionalized biological device design when considering obtaining the antibacterial properties and hemocompatibility simultaneously. With further systematic evaluation on the biochemical and physical function at the interface between graphene and organism, the practical application of Ge-based biological device can be greatly promoted.

## Methods

### Graphene deposition on Ge surface

The graphene films were deposited on the Ge (110) substrate by atmospheric pressure chemical vapor deposition (APCVD). Ge substrate was cut into small pieces and put in an inner quartz processing tube (50 nm in diameter) located at the center of horizontal quartz tube. Then the quartz tube was evacuated to approximately 0.01 Pa and followed by filling with gas mixture comprised of 200 standard cubic cm per min (sccm) argon (Ar, 99.9999% purity) and 50 sccm hydrogen (H_2_, 99.9999% purity). After heating to the desired temperature (910 °C), methane was introduced to deposit graphene films for various durations ranging from 100 min to 200 min. After deposition, the supply of methane was switched off and the furnace was cooled down to room temperature under the mixture of flowing H_2_ and Ar gas.

### Surface characterization

The surface morphology was characterized by atomic force microscope (AFM Bruker Multimode 8 system) in tapping mode and scanning electronic microscopy (SEM Zeiss Supra 55) with an acceleration voltage of 3 keV. Raman spectroscopy (HORIBA Jobin Yvon HR800) was utilized to evaluate the quality and uniformity of the CVD-grown graphene film. The analysis of the X-ray photoelectron spectroscopy (XPS) was carried out by using a Gaussian–Lorentzian peak shape after removing background spectra by Shirley algorithm.

### Potentiodynamic polarization curve test

A typical system consisting of three electrodes was used to conduct the electrochemical test. The graphite rod, saturated calomel electrode and the test sample were used as a counter electrode, reference electrode and working electrode, respectively. The physiological saline (0.9 wt% NaCl solution) was used as the electrolyte during the electrochemical test. The scanning parameter of the electrochemical workstation (CHI760C) was controlled at 1 mV/s from the cathode to the anode direction.

### Antibacterial ability assessment

A LIVE/DEAD^®^Baclight^TM^ Bacterial Viability Kit (L13152, Molecular probes) was used to implement fluorescence staining to reveal the viability of bacteria on the samples, and then the fluorescence staining results were observed by fluorescence microscopy (Olympus GX71). This kit contains a mixture of a SYTO^®^9 green fluorescence nucleic acid stain and red fluorescence nucleic acid stain, propidium iodide (PI).

For scanning electron microscope (SEM) observation, 60 μL bacterium solution with the concentration of 10^7^ CFU mL^−1^ was seed on the sample surface, incubated at 37 °C for 24 h, fixed by 2.5 v% glutaraldehyde solution for night at 4 °C and then dehydrated by graded ethanol (30, 50, 75, 90, 95, and 100 v/v%) for 10 min each. Subsequently, samples were finally dried by hexamethyldisilazane ethanol solution series followed by coating platinum before SEM observation.

### Hemocompatibility evaluation

To evaluate the hemolysis rate (HR) of the samples, fresh human whole blood was obtained from the healthy donors. The blood collecting procedures were performed in compliance with institutional guidelines approved by Zhongshan Hospital, Shanghai, and the experiments were conducted according to National standard of China GB/T 16886.4-2003 protocol under the condition of donors’ consent. All samples were sterilized by 75 v/v% ethanol solution and blow-dried. Then samples were rinsed with physiological saline and then immersed in physiological saline at 37 °C for 30 min. Meanwhile, untreated physiological saline and ultrapure water were utilized as the negative and positive controls respectively. Subsequently, diluted human whole blood was add to each well and incubated at 37 °C for another 1 h. Afterward, the mixture solution were centrifuged for 5 min at 3000 rpm. Finally, supernatant were measured at 545 nm by Multimode Reader (Biotech Cytation 5), and HR can be acquired by the equation as follows^44^:


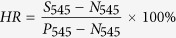


the absorbance for samples, negative control and positive control were represented by S_545_, N_545_ and P_545._ respectively.

Platelet rich plasma (PRP) was obtained by centrifugation of human whole blood for 15 min at 1500 rpm. The samples was sterilized and dried as mentioned above, and then incubated for 30 min. Soon afterward, 50 μL PRP was seeded on each surface of the samples and incubated for another 2 h. After rinsed with PBS (3 times, 5 min each), samples were fixed by immersing in 2.5 v% glutaraldehyde solution for night at 4 °C. In order to observe the microstructure morphology, the platelets on the samples were dehydrated by the same processing conditions as mentioned in section of Antibacterial ability assessment.

## Additional Information

**How to cite this article**: Geng, H. *et al.* Antibacterial ability and hemocompatibility of graphene functionalized germanium. *Sci. Rep.* **6**, 37474; doi: 10.1038/srep37474 (2016).

**Publisher's note:** Springer Nature remains neutral with regard to jurisdictional claims in published maps and institutional affiliations.

## Supplementary Material

Supplementary Information
