# Supplementary material for: Antibacterial ability and hemocompatibility of graphene functionalized germanium
Source: Sci Rep. 2016 Nov 23;6:37474. doi: 10.1038/srep37474 (PMC5120343; doi:10.1038/srep37474)
Supplement: Supplementary Information [file srep37474-s1.pdf]

Supplementary Information

for

Antibacterial ability and hemocompatibility of graphene functionalized germanium

Hao Geng[§a,c], Jiayun Dai[§b,c], Jinhua Li[a,c], Zengfeng Di*[b] and Xuanyong Liu*[a]

[a] State Key Laboratory of High Performance Ceramics and Superfine Microstructure, Shanghai Institute of Ceramics, Chinese Academy of Sciences, Shanghai 200050,China.

[b] State Key Laboratory of Functional Materials for Informatics, Shanghai Institute of Microsystem and Information Technology, Chinese Academy of Sciences, Shanghai 200050, China.

[c] University of Chinese Academy of Sciences, Beijing 100049,China.

[§] These authors contributed equally to this work

[*]Corresponding Author:

Prof. Xuanyong Liu

State Key Laboratory of High Performance Ceramics and Superfine Microstructure, Shanghai Institute of Ceramics, Chinese Academy of Sciences, Shanghai 200050, China

E-mail: xyliu@mail.sic.ac.cn

Tel: 86-21-52412409

Prof. Zengfeng Di

State Key Laboratory of Functional Materials for Informatics, Shanghai Institute of Microsystem and Information Technology, Chinese Academy of Sciences, Shanghai 200050, China

E-mail: zfdi@mail.sim.ac.cn

Tel: 86-21-62511070

Results

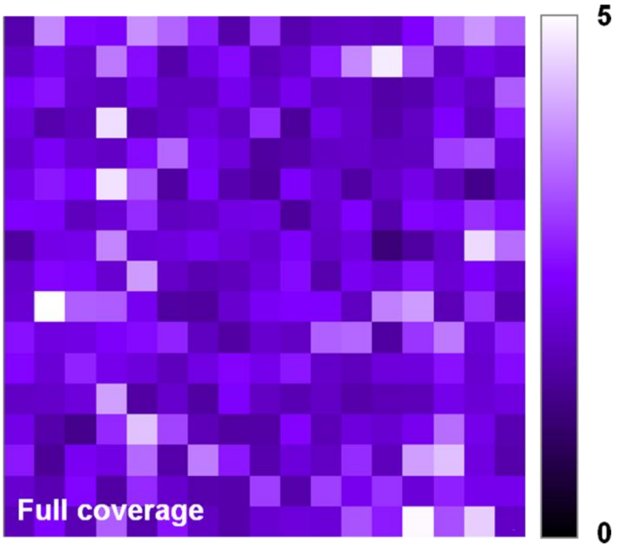

Fig.S1 Raman mapping of Full-Gr (testing area:50×50 μm)

Raman mapping was used to further confirm the layer number and uniformity of the obtained Full-Gr sample. As shown in Fig.S1, the mean ratio of I_{2D}/I_G is more than 1.5, which indicated that the obtained graphene film is monolayer [1].

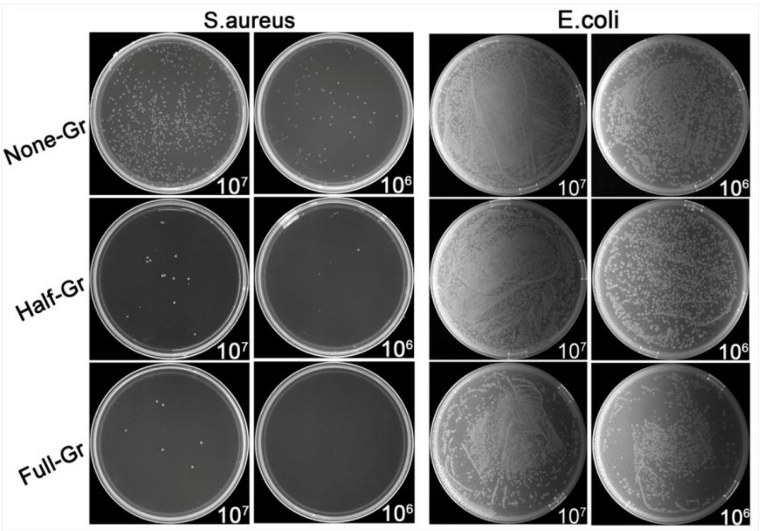

Fig.S2 Typical photographs of re-cultivated S.aureus strain (left panel) and E.coli strain (right panel) on agar culture plate with the seeded concentrations of detached

bacteria being 10^7 and 10^6 CFU mL^{-1}, respectively.

The re-cultivated S.aureus scatters in the form of independent colonies other than the spreading velum of re-cultivated E.coli at a higher concentration. Meanwhile, few number of S.aureus colonies compared to serried E.coli colonies are apparently on the agar plate at a lower concentration, it can be speculated that the Ge itself behaves a better antibacterial ability on the S.aureus with respect to E.coli.

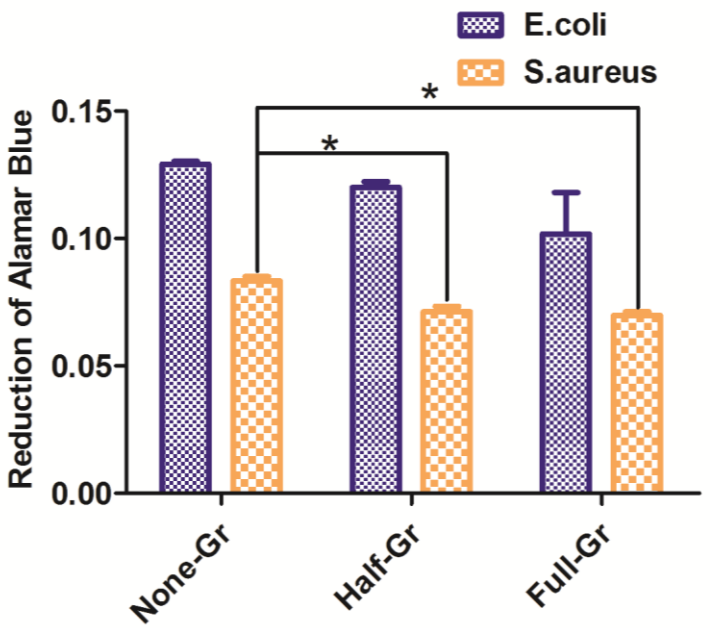

Fig.S3 Reduction of Alamar blue after re-cultivating with the detached bacteria solution with the concentration of 10^7 $CFUmL^{-1}$ for 4 h. $*P < 0.05$

Alamar blue, a kind of nonpoisonous dyestuff, which is commonly used to evaluate the cell proliferation behavior, can also be employed to assess the proliferation ability of bacteria as well as viability of bacteria [2]. The detached dilute

bacterium solution was mixed with Alamar blue with a volume ratio of 9 for 4 h at 37 ℃, the absorbance was measured at 570 nm and 600 nm by using Multimode Reader (Biotech Cytation 5). As shown in Fig.S3, once the surface of Ge is coated with graphene films, no matter what extent of the coverage area, significant decrease could be seen in the reduction rate of Alamar blue with respect to None-Gr substrate, which indicates that the poor surviving status and weak proliferation ability of the detached S.aureus bacterium solution from the graphene film covered specimen. Nevertheless, in the terms of the E.coli, the reduction rate of Alamar blue shows an inconspicuous declining trend accompanied with the increase of the coverage area of the graphene films on the Ge substrate. All these results visualize a favorable consistence with the characterization results of agar culture plate.

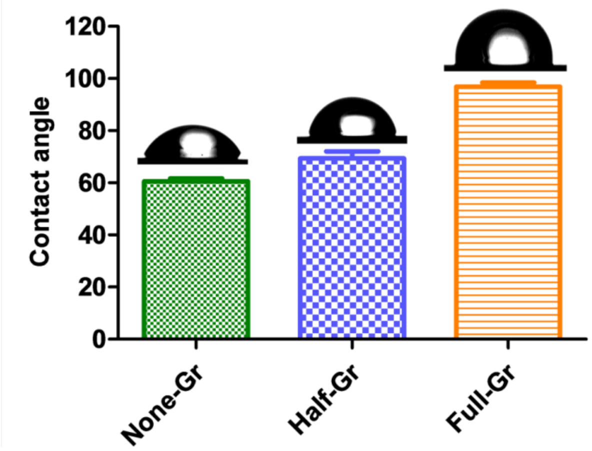

Fig.S4 Variation of the water static contact angle along with the coverage area change of graphene.

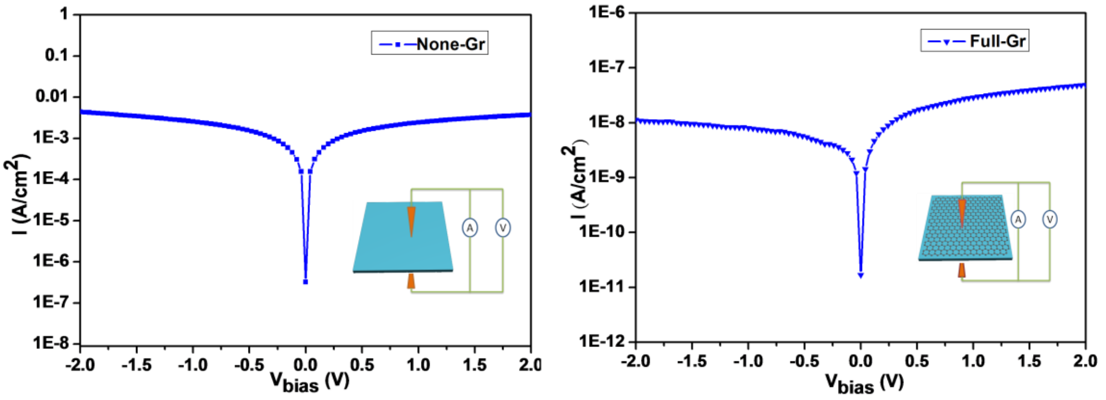

Fig.S5 Typical Current-Voltage (I-V) characteristic curve of Ge (left) and graphene@Ge junction (right). Schematic diagrams for IV testing are provided as insets. The on/off ratio of graphene@Ge junction was about 48.

Experimental Methods

Agar culture plate test. The effect of the different coverage area of graphene films on the antibacterial properties of Ge substrate was evaluated by drop-test of experimental strains which including S.aureus as the gram-positive strain and E.coli as the gram-negative strain. After sterilization in 75 v/v% ethanol solution and dry, a droplet of strain solution with the concentration of 10^7 CFU mL^{-1} was seed on the sample surface to a density of 60 μL cm^{-2} and then incubated at 37 ℃ for 24 h. The detached bacterium solution was diluted and evenly inoculated onto the standard agar culture plate for incubating another 16 h at 37 ℃. Subsequently, the culture plates were photographed according to the National Standard of China GB/T 4789.2 protocol.

Alamar blue test of bacteria viability. A droplet of strain solution with the

concentration of 10^7 CFU/mL was seed on the sample surface to a density of 60 μL cm^{-2} and then incubated at 37 ℃ for 24 h. The detached bacterium solution was diluted 10-folds to concentration of 10^6 CFU/mL by using physiological saline. Subsequently, the detached dilute bacterium solution was mixed with Alamar blue with a volume ratio of 9 for 4 h at 37 ℃, the absorbance was measured at 570 nm and 600 nm by using Multimode Reader (Biotech Cytation 5).

Current–voltage curve test (I-V test). The *I-V* data were collected under ambient conditions using an Agilent (B1500A) semiconductor parameter analyzer.

Statistical analysis. Statistically significant differences (P) between the various groups were measured using one-way analysis of variance and Tukey's multiple comparison tests by GraphPad Prism 5 statistical software package. The data are expressed as means standard deviation (SD).

References

1. Ferrari, A. C. et al. Raman spectrum of graphene and graphene layers. *Phys Rev Lett* **97** (2006).
2. Nakayama, G. R., Caton, M. C., Nova, M. P. & Parandoosh, Z. Assessment of the Alamar Blue assay for cellular growth and viability in vitro. *J Immunol Methods* **204**, 205-208 (1997).
